# Transparent Exploration of Machine Learning for Biomarker Discovery from Proteomics and Omics Data

**DOI:** 10.1021/acs.jproteome.2c00473

**Published:** 2022-11-25

**Authors:** Furkan M. Torun, Sebastian Virreira Winter, Sophia Doll, Felix M. Riese, Artem Vorobyev, Johannes B. Mueller-Reif, Philipp E. Geyer, Maximilian T. Strauss

**Affiliations:** † OmicEra Diagnostics GmbH, 82152 Planegg, Germany; ‡ Novo Nordisk Foundation Center for Protein Research, University of Copenhagen, 2200 Copenhagen, Denmark

**Keywords:** machine learning, mass spectrometry, diagnostics, omics, proteome, metabolome, transcriptome

## Abstract

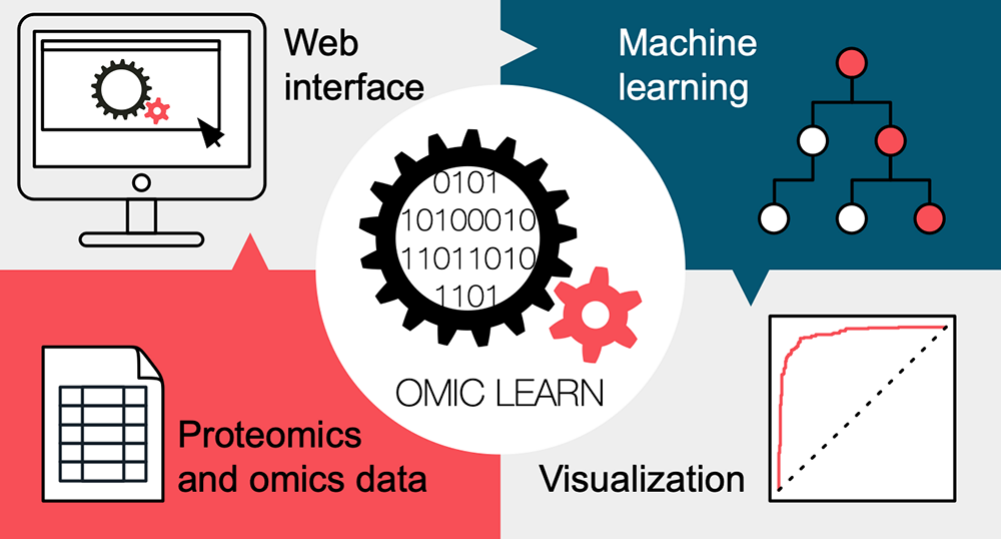

Biomarkers are of central importance for assessing the health state and to guide medical interventions and their efficacy; still, they are lacking for most diseases. Mass spectrometry (MS)-based proteomics is a powerful technology for biomarker discovery but requires sophisticated bioinformatics to identify robust patterns. Machine learning (ML) has become a promising tool for this purpose. However, it is sometimes applied in an opaque manner and generally requires specialized knowledge. To enable easy access to ML for biomarker discovery without any programming or bioinformatics skills, we developed “OmicLearn” (http://OmicLearn.org), an open-source browser-based ML tool using the latest advances in the Python ML ecosystem. Data matrices from omics experiments are easily uploaded to an online or a locally installed web server. OmicLearn enables rapid exploration of the suitability of various ML algorithms for the experimental data sets. It fosters open science via transparent assessment of state-of-the-art algorithms in a standardized format for proteomics and other omics sciences.

## Introduction

Machine learning (ML) is one of the most exciting opportunities for transforming scientific discovery today. While ML and its first algorithms were conceptualized decades ago, increasing computational power and larger data sets have now clearly demonstrated the superiority of ML approaches over classical statistical methods in many applications. Concurrently, advances in omics technologies have enabled the generation of large and complex biological data sets from the analysis of hundreds to thousands of samples (in some cases stemming from hundreds of individuals),^1−4^ which now allows ML to extract meaningful biological information from the data. This also applies to mass spectrometry (MS)-based proteomics, which has become the method of choice for the quantitative investigation of the entirety of proteins and their modifications in a biological system.^5−8^ Continuous technological advances transform MS-based proteomics from a basic research tool to a powerful clinical technology. As technological challenges in robustness, throughput, and reproducibility are being solved, MS-based proteomics is becoming increasingly popular for the analysis of clinical samples and an ideal tool for biomarker discovery. The development of automated sample preparation pipelines and increasingly robust liquid chromatography (LC) and MS systems enable the analysis of large studies. Such large data sets are challenging to analyze in conventional ways but are well-suited to ML algorithms, which can identify promising protein signatures and predict physiological states based on proteome data and additional clinical metadata. Recently, we applied ML in studies comprising hundreds of cerebrospinal fluid (CSF) or urine samples to predict the manifestation of neurodegenerative diseases.^9,10^ In these projects, established biomarkers associated with the investigated diseases ranked among the top candidates such as tau, SOD1, and PARK7 in Alzheimer’s Disease (AD) and VGF and ENPEP in Parkinson’s Disease (PD), and potential novel ones were uncovered.

For experimental researchers, applying ML to proteomics and other omics data sets requires adapting existing tools to the task at hand. Multiple commercial frameworks are targeted toward general ML applications, such as RapidMiner and KNIME,^11^ which typically have a free tier for academics. Commercial cloud providers such as AWS, Google, and Microsoft Azure have customized ML products, often tailored for big data applications. Latest research and competitive machine learning on platforms like Kaggle use popular packages such as scikit-learn or XGBoost that allow predictive data analysis in principle, but researchers still require programming knowledge to write their own ML pipelines.^12,13^ In particular, the currently available packages in Python or R require writing a data pipeline with code since they typically have no graphical interface. A noteworthy exception is the Galaxy project, a server-based scientific workflow system that aims to make computational biology more accessible.^14^ Another widely used tool is Weka, a collection of machine learning algorithms for data mining tasks in Java with a one-click installation and graphical user interface.^15^

When wanting to reproduce published results, the same software environment needs to be set up and configured with the matching package versions and random seeds. Especially in ML, selecting the appropriate methods is far from obvious to the nonspecialist. Moreover, many parameters can be altered to tune the algorithms, which might change from version to version, resulting in reproducibility issues. While several packages exist that perform automatic optimization of parameters and provide a “best” solution, manual verification and benchmarking of algorithms are limited. This restricts understanding of the role of the data and the algorithm in the model.

Additionally, omics sciences and ML require special domain knowledge as metrics can be deceiving, and algorithms might need special preselection or preprocessing steps. For instance, in some studies, the receiver operating characteristics (ROC) curve might be useful to confirm the performance, while precision-recall (PR) curves are mandatory in imbalanced data sets.^16^ Imbalanced data sets refer to data sets where one group is overrepresented, which can cause misleading performance metrics. Thus, transparent and open-source software would be favorable, particularly in the interest of open and reproducible science.^17^

To address these issues and to make machine learning more accessible to support the current initiatives on biomarker discovery, we here introduce OmicLearn, a ready-to-use ML web application specifically developed for omics data sets. We describe OmicLearn’s architecture and show its benefits by applying it to a recently published proteomics study investigating alterations in the CSF of AD patients.^9^ OmicLearn incorporates community efforts by building on scientific Python libraries and is available as open-source. It can be accessed via the hosted web server or downloaded for local deployment. We additionally provide a one-click installer for Windows, Mac, and Linux.

To provide a perspective on the utility of OmicLearn, we briefly discuss already published studies that used OmicLearn. Geyer et al. measured the serum proteome of PCR-negative controls and hospitalized COVID-19 patients. In addition to statistical analysis with established methods such as significance tests and representation as volcano plots, they utilized OmicLearn to evaluate how well ML-based classification would distinguish patients from controls. OmicLearn repeatedly split the data into different subsets, trained a classifier, and provided performance metrics. The resulting ROC curve had an average area under the curve (AUC) of 0.90 ± 0.08, and the PR curve had an average AUC of 0.92 ± 0.06.^18^

Karayel et al. performed proteome profiling of CSF to study PD in two cohorts and could identify a small number of commonly altered proteins.^19^ Next, they combined both cohorts and used OmicLearn to apply ML for classification. The resulting ROC curve had an average AUC of 0.72 ± 0.08. Interestingly, the most important feature for the classifiers was prolactin, which was not significantly regulated in either cohort, highlighting the potential of the ML approach.

## Materials and Methods

### Overview of the OmicLearn Architecture

**Figure 1:**
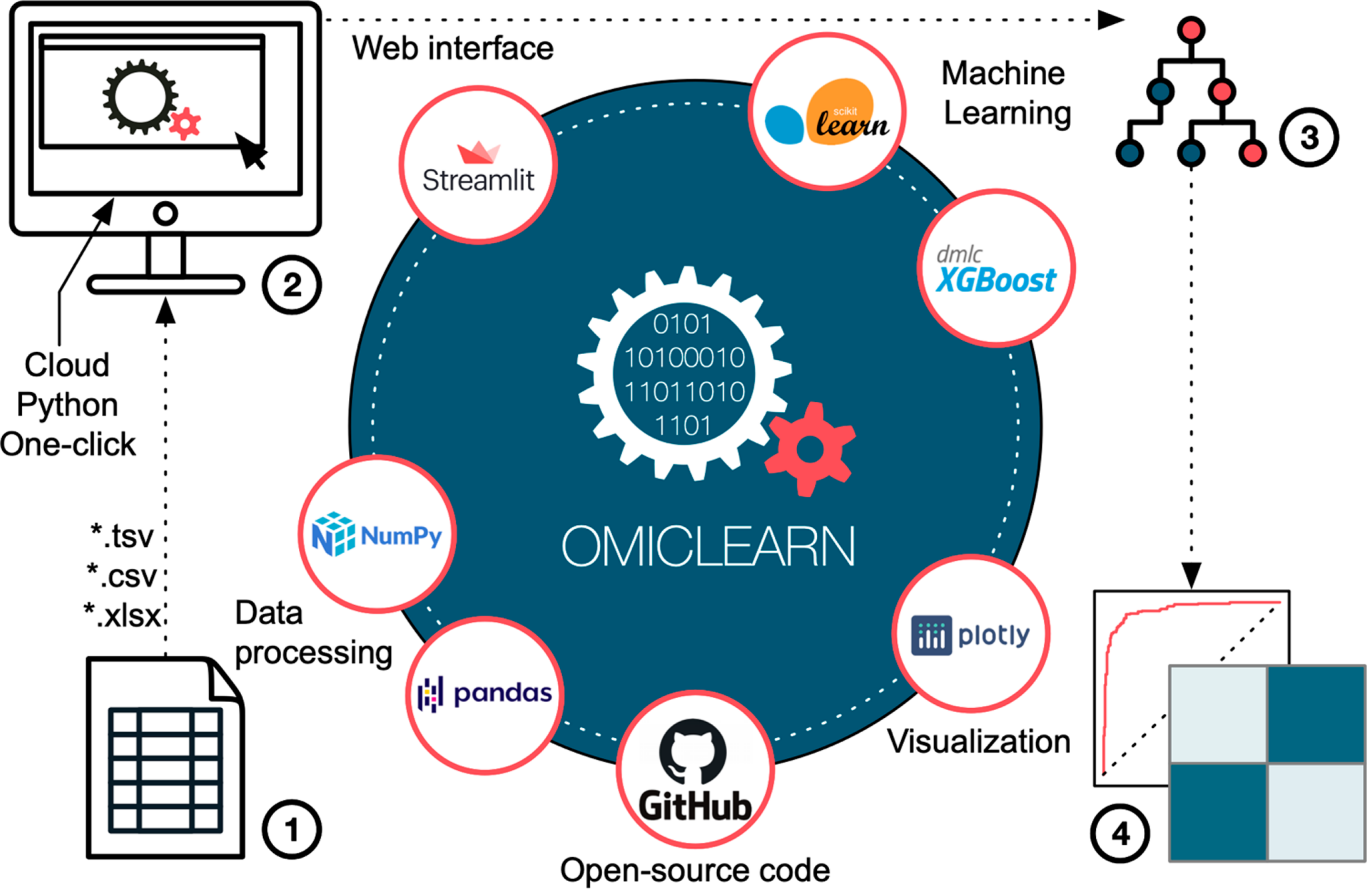
OmicLearn architecture. Left side: tabular experimental data files can be uploaded to OmicLearn as *.tsv, *.csv, or *.xlsx (Excel format). (1) Internally, OmicLearn uses the NumPy and pandas packages to import and handle data. OmicLearn is an interactive web-based tool built on the Streamlit package (2), which can be used to explore the data interactively. The application can be installed via a one-click installer or accessed online so that it is readily accessible for nonexperts. Right side: OmicLearn has access to the large machine learning libraries of scikit-learn and additional algorithms such as XGBoost. (3) The pipeline is set up to perform classification tasks on omics data sets with multiple cross-validations of results. Various performance metrics are displayed, leveraging the Plotly library. (4) The OmicLearn repository is hosted on GitHub and is open-source. Logos courtesy of the respective library/company (streamlit.io, scikit-learn, xgboost, plotly, github.com, pandas, and NumPy).

OmicLearn consists of a central web interface, an analysis core, and visualization (Figure 1). Within the analysis core, data processing builds on open-source data manipulation tools such as pandas^20^ and NumPy,^21^ specifically designed for multidimensional matrices and arrays. To implement state-of-the-art ML and preprocessing methods, we built OmicLearn on scikit-learn and advanced machine learning algorithms such as XGBoost (eXtreme Gradient Boosting). Scikit-learn is a widely used library for classification, regression, and clustering problems, which incorporates standard preprocessing, feature selection, and cross-validation techniques needed in ML.^12^ XGBoost comes with additional algorithms, improved performance, and an optimized memory usage.^13^

The interactive web interface and visualization components are built on the recently developed but already extremely popular open-source framework Streamlit (https://www.streamlit.io). Dropdown menus allow the straightforward definition of data set specific variables and the selection of various parameters for different ML algorithms. A core feature that facilitates usage, especially for novice users, is the automated interface update based on previously made selections, preventing invalid choices. To illustrate this further, when a user uploads a data set with missing values, a warning will be displayed that imputation is required or that a classifier that supports missing values needs to be selected. Here, imputation of missing values refers to the practice of replacing signals that were not detected by the experiment with reasonable estimates (e.g., the mean intensity of a protein in the study) so that samples can be compared. Theoretically, in this case, a user could make an invalid selection by not selecting missing value imputation and choosing an incompatible classifier. However, OmicLearn prevents this on the interface level: If the missing value imputation is set to none and the data set has missing values, the number of selectable classifiers is reduced to compatible ones.

Results are visualized with the graphic Python library Plotly (https://plotly.com/python) to generate high-quality interactive graphs, which can be exported as *.pdf, *.png, or *.svg. For guidance, we implemented a ReadtheDocs documentation (https://omiclearn.readthedocs.io/en/latest/) that provides background knowledge about OmicLearn, its ML algorithms, and the available methods. Additionally, the documentation supplies information on clinical MS-based proteomics, recommendations, an installation guide, and a user manual for the tool. For in-depth information about the available selections, we directly link their headers to the documentation of scikit-learn.

The code of OmicLearn is released as open-source under the Apache License (2.0). The tool is available on GitHub https://github.com/MannLabs/OmicLearn, which includes the documentation, the complete source code, and the example data set described below. The example data set can be used to explore the platform without uploading a data set and reproduce the results presented here. OmicLearn can be installed and run locally to enable use in restricted or sensitive environments. To facilitate installation, we include a one-click installer for Windows, Linux, and Mac. Alternatively, a running instance of the online app can be accessed via the website http://OmicLearn.org. Here, we use the sharing option provided by Streamlit.

## Results

### Using OmicLearn

Data sets can be uploaded via drag and drop or browsing a local drive. Internally, OmicLearn is built on the widely used pandas and NumPy packages to import and store data. Data sets should be supplied in a .tsv, .csv, or .xlsx format, typical output formats of packages such as MaxQuant, DIA-NN, or AlphaPept.^22−24^ The data sets need to meet distinct criteria with regard to the structure of the data matrix. Each row should correspond to a sample, each column to a feature to be used for classification, and every column must have a header. Features can be supplied as two types: main and additional. Main features typically comprise the abundance information on every analyte (e.g., protein or metabolite intensities), while additional features are associated with clinical information such as age, sex, or disease status of the samples or subjects. To provide an intuitive way to use additional features and distinguish them from main features, OmicLearn requires their column names to start with an underscore “_” (e.g., “_age”). Ultimately, this allows researchers to quickly assemble matching data matrices with text or spreadsheet manipulation tools to be used with OmicLearn. As additional metadata is often provided in various formats and is typically not integrated into the search result output, we further provide detailed documentation on how to use and format the output files for the aforementioned search engines.

To quickly test out the features of OmicLearn without uploading a custom file, we provide a tutorial sample file and a real-world data set from a recently published study on biomarker discovery in AD using CSF.^9^

**Figure 2:**
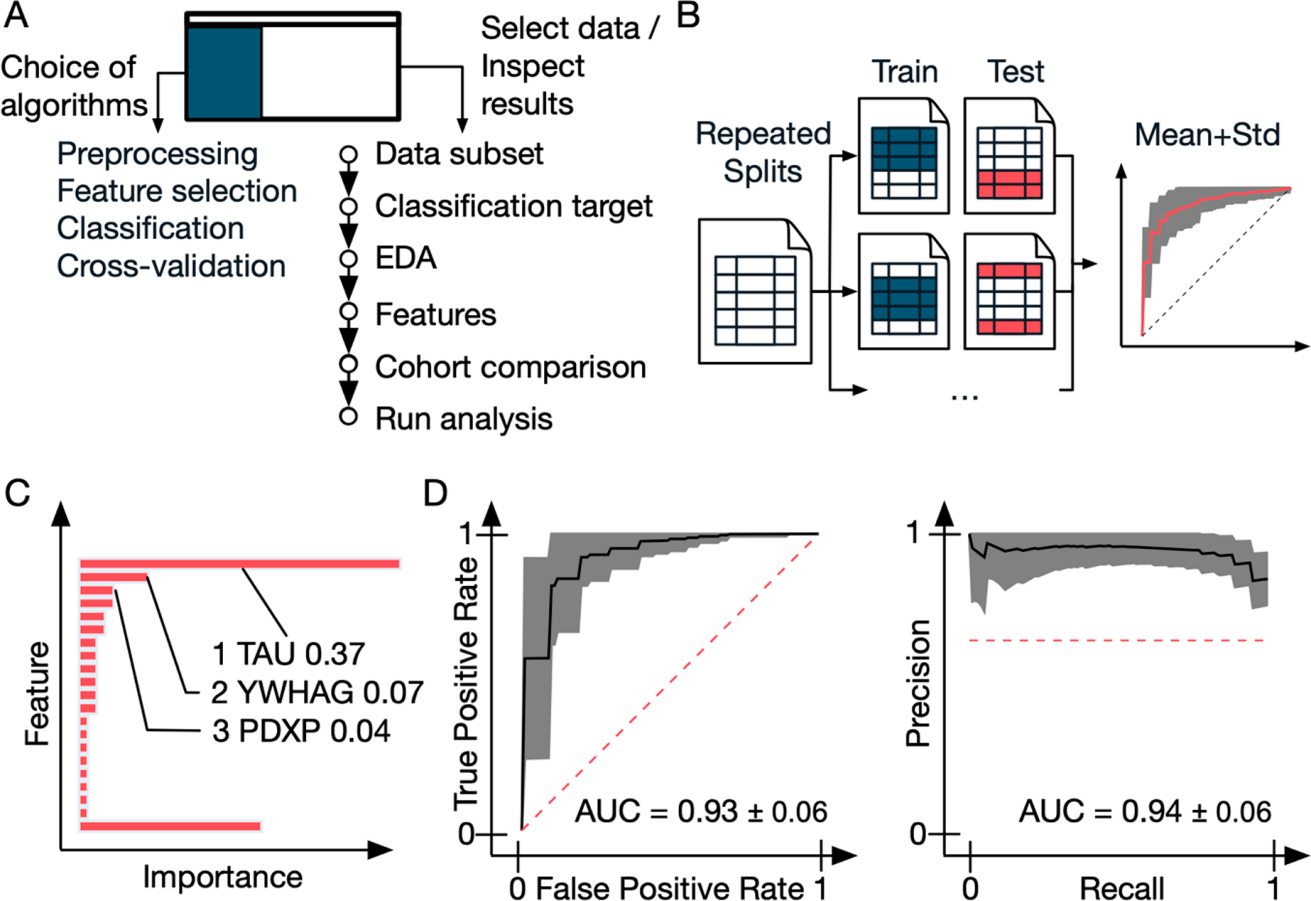
Functional flow of OmicLearn and example performance metrics. (A) The OmicLearn’s landing page is composed of two functional elements. The left side allows setting the options for the ML pipeline such as selecting the ML classifier and setting algorithmic parameters. The right side allows manipulating the data set and exploration of results. The process is interactive and follows a linear flow, e.g., whenever an option is selected only choices that will match the previously selected parameters will be shown. (B) Cross-validation (CV) strategy: data is repeatedly split into train and validation sets so that means and standard deviations can be estimated. (C) Feature importance: this plot shows the feature importance from the ML classifier, averaged over all classification runs. The definition of the feature importance depends on the used classifier, e.g., for a LogisticRegression it is the weights of the linear model. The annotation on the *y*-axis is interactive and will directly link to a search request on NCBI. Highlighted is tau, which was found as one of the most important features in the underlying Alzheimer’s study. Note that the feature importance is sorted by magnitude; the lowest bar is the remainder of all features not shown. (D) Interactive receiver operating characteristics (ROC) and precision-recall (PR) curve: The ROC curve shows individual CV splits as well as an average ROC curve with a confidence interval. The PR curve shows individual CV splits as well as an average PR curve with a confidence interval.

Once a file is uploaded, OmicLearn’s ML interface appears, consisting of two separate selection menus for ML options and for data set specific feature definitions (Figure 2A). The core steps of the pipeline can be found in the left sidebar, where the user can specify individual parameters for random state, preprocessing, feature selection, classification, and cross-validation. As an example, OmicLearn offers the choice between several algorithms for classification, including AdaBoost, Logistic Regression, Random Forest, XGBoost, Decision Tree, KNN Classification, and linear support vector classification (briefly described with additional links in the OmicLearn documentation). Within the interactive interface, several hyperparameters can be defined according to the chosen model or algorithm. In an ML context, a hyperparameter is a parameter that can be used to control the learning process of an algorithm. Furthermore, a random state slider allows the specification of a seed state to make random operations such as train-test splits deterministic to ensure reproducibility of the predictions.

The underlined headlines of the ML options such as “Feature selection” are linked to the documentation. Here, we supply a stepwise manual to apply OmicLearn and more information for all sections and methods. Moreover, the user will find references for supporting information for the ML algorithms, metrics, and scores. Table 1 shows a summary of the currently implemented options for each processing step. We want to highlight that it is, in principle, very difficult to recommend a general best-practice algorithm that will achieve the best performance as this will strongly depend on the data set and the task in mind. A more informed decision on when to use which algorithm would require expert knowledge of the underlying algorithmic details. Here, OmicLearn is intended to rather provide insight into how the different algorithms will affect performance or to reproduce existing results with defined hyperparameters. As iterating through different algorithms typically requires only a couple of seconds, we encourage the user to try different algorithms and compare the results with the “Session history” section.

**Table 1:** Currently Implemented Options for Each Processing Step in OmicLearn

step	options
EDA	principal component analysis (PCA)
	hierarchical clustering
preprocessing	StandardScaler
	MinMaxScaler
	RobustScaler
	PowerTransformer
	QuantileTransformer
feature selection	ExtraTrees
	k-best (mutual_info_classif)
	k-best (f_classif)
	k-best (chi2)
classification	AdaBoost
	LogisticRegression
	KneighborsClassifier
	RandomForest
	DecisionTree
	LinearSVC
	XGBoost
cross-validation	RepeatedStratifiedKFold
	StratifiedKFold
	StratifiedShuffleSplit

Subsets of uploaded data sets can be created based on an additional feature column, e.g., when having a multicenter study and only wanting to train on the data of a specific study center.

Within the “Classification target” section, the user can specify the column that contains the classification target. Here, they can define two classes that are based on unique values within this column that the classifier will be trained to distinguish. In this context, a class refers to the groups that the classifier should learn to separate. In a typical setup, this could be the disease state to distinguish patient and control samples. If there are more than two unique values, each class can be defined to consist of multiple values, or values can be excluded when training the classification algorithm.

While thorough exploratory data analysis (EDA) should be done before applying the machine learning layer with OmicLearn, we provide utility functions to perform basic EDA on the uploaded data set. This includes principal component analysis (PCA) and hierarchical clustering to identify potential artifacts in the data set that could lead to unreliable performance metrics. OmicLearn also allows one to include additional feature columns in the classification. This refers to features that are not main features (i.e., protein intensities) but additional metadata, such as age or clinical parameters. The usage of these features can be selected under the “Additional features” section. If a column contains non-numerical data such as “condition_a”, “condition_b”, and “condition_c” for a category, OmicLearn will convert the values to numerical data such as 0, 1, and 2. In this section, users might upload their *.csv file (comma “,” separated), where each row corresponds to a feature to be excluded.

Furthermore, it is possible to manually select the main features via “Manually select features”. This feature is intended to explore how a classifier performs when a defined set of features is provided. While this can be useful to investigate individual proteins it is to note that this could lead to biased results, when applied incorrectly. An example case would be when first testing and extracting for regulated proteins, and then only selecting them as features. Effectively, this would leak information from the test set and make the performance metrics less reliable, as described in the literature.^25^ This effect will not occur in the default settings for OmicLearn. Here, the automatic feature selection step is not applied on the entirety of the data set but only on the respective cross-validation split.

Lastly, the option “Cohort comparison” allows using one of the additional feature columns to split the data set into different cohorts to train on one cohort and test on the other. Once all parameters are set, clicking on the “Run analysis” button will initiate the selection of the best features and calculation of the predictive model.

### Interpretation of Results

OmicLearn reports various metrics, ranging from reports on important features to the evaluation of the applied ML models. These results are displayed in several tables and graphs. A bar plot ranks the features with the highest contribution to the prediction model (20 in our tutorial data set; Figure 2C) from all of the cross-validation (CV) runs. For instance, in our sample data set analysis, the known biomarker tau (P10636) displayed the highest feature importance value, as described in the original study. This information is also available as tables in *.csv format. To comfortably retrieve more knowledge about these features, we directly linked their IDs or names to a National Center for Biotechnology Information (NCBI) search.

In order to evaluate the performance of an ML model, a study needs to be split into train, validation, and holdout (test) sets. Optimization is performed using the training and validation sets, and the model that is ultimately used is being evaluated using the unseen holdout set. As already mentioned, OmicLearn is intended to be an exploratory tool to assess the performance of algorithms when applied to specific data sets at hand, rather than a classification model for production. Therefore, no holdout set is used, and the performance metrics have to be interpreted accordingly. This also prevents repeated analysis of the same data set and choosing the same holdout set from leading to a selection bias and consequent overinterpretation of the model.

The strategy of splitting data is crucial to overcome the common ML problems of over- or underfitting. Overfitting occurs when applying a model with high complexity that learns on unrelated noise. Overfitted models will be capable of describing the sample with high accuracy but will not generalize well when validating another data set. In our context, this is frequently observed when study-specific biases are present that are not found in future observations. Underfitting happens when the model is not sufficiently complex and is, therefore, not capable of learning the subtleties of the sample characteristics, resulting in suboptimal performance. Even though the throughput of omics sciences is rapidly increasing, the number of analyzed samples is generally small compared to the number of features that can be measured. To illustrate, a sample cohort may be in the range of hundreds but we are measuring thousands of proteins, making ML particularly prone to overfitting. In order to use the existing data most efficiently, we use CV, in which data is repeatedly split into train and validation sets (RepeatedStratifiedKFold method). For this purpose, we integrated a stratified splitting technique, meaning that the original class ratio will be preserved for the splits. OmicLearn offers additional split methods such as StratifiedKFold and StratifiedShuffleSplit, which can be selected in the ML options (Figure 2B). These measures aim to prevent misleading models that learned on biased distributions. To give an example, this could be the case for a data set where 1% of the patients have a rare condition and random splits do not contain data points with the condition. The model could learn to always predict the majority class and would reach 99% accuracy.

The number of features that are being used for the model can be either selected by the user or automatically selected with feature selection algorithms built into OmicLearn. The feature importance scores obtained from the classifier after all CV runs are displayed in a horizontal interactive bar chart and an exportable table (Figure 2C). The feature importance is additionally provided in tabular format and contains the standard deviations. The meaning of the quantity feature importance depends on the underlying classifier, e.g., for a LogisticRegression it would be the weights of the linear model, while for a DecisionTree model, it would be the Gini importance. More information can be found when following the links to each classifier in the Wiki.

The feature selection is applied for each split during the CV process so that no information leakage occurs.

We further implemented ROC for a graphical representation of model performance (Figure 2D). They display the true positive rate (sensitivity) against the false positive rate (1 – specificity) in an easily interpretable form. In the supplied plot, the mean ROC curve (black) is displayed together with the standard deviation (gray background) of the different curves from the various train and validation set splits. The area under the curve receiver operating characteristics (AUC-ROC) is a numerical value to assess the prediction; it would be 1.0 in the case of perfect discrimination. In addition, we use PR curves displaying the sensitivity (recall) against the positive predictive value (Figure 2D). PR curves are valuable for performance assessments, especially when dealing with imbalanced data sets, where one class is more frequent than the other.^26^ To further evaluate the quality of the predictions, we supply a 2 × 2 “confusion matrix” to compare predicted and actual classes. A confusion matrix is a table that compares the actual condition to the predicted condition for the classes of the classifier. In the sample data set, it displays the number of correctly predicted positive and negative AD patients as well as the number of false-positive and false-negative predictions.

The overview of all results is available in one comprehensive table in the “Results” section. We further provide a publication-ready summary text for describing packages, libraries, methods, and parameters. Finally, since researchers might perform multiple runs in OmicLearn to explore different learning conditions, previous results are listed in the “Session History” section. In this way, users can easily compare current with previous results. Additionally, a download option for the session history as *.csv exists. The graphics generated by OmicLearn can be saved in a publication-ready format such as *.pdf, *.svg, and *.png, and all tables are available as .csv files.

For further validation of the achieved results, the OmicLearn documentation contains a recommendations page with potential pitfalls (e.g., artifacts, misuse of settings, or guidelines on sample size).

### Application Examples

The underlying type of an ML classifier can have a drastic effect on the model performance depending on the given data set it is applied to. Therefore, models should be selected to fit the nature of the problem. In the analysis of our sample AD data set with OmicLearn, we quickly evaluated seven ML algorithms. For this, we selected the Alzheimer data set and set the classification target to the clinical AD diagnosis and “_gender” as an additional feature. Next, we did run the analysis using the default settings. Subsequently, we changed only the classifier and reran the analysis. Within the user interface, this involves only changing the selected classifier in the dropdown menu and again pressing the Run analysis button.

**Figure 3:**
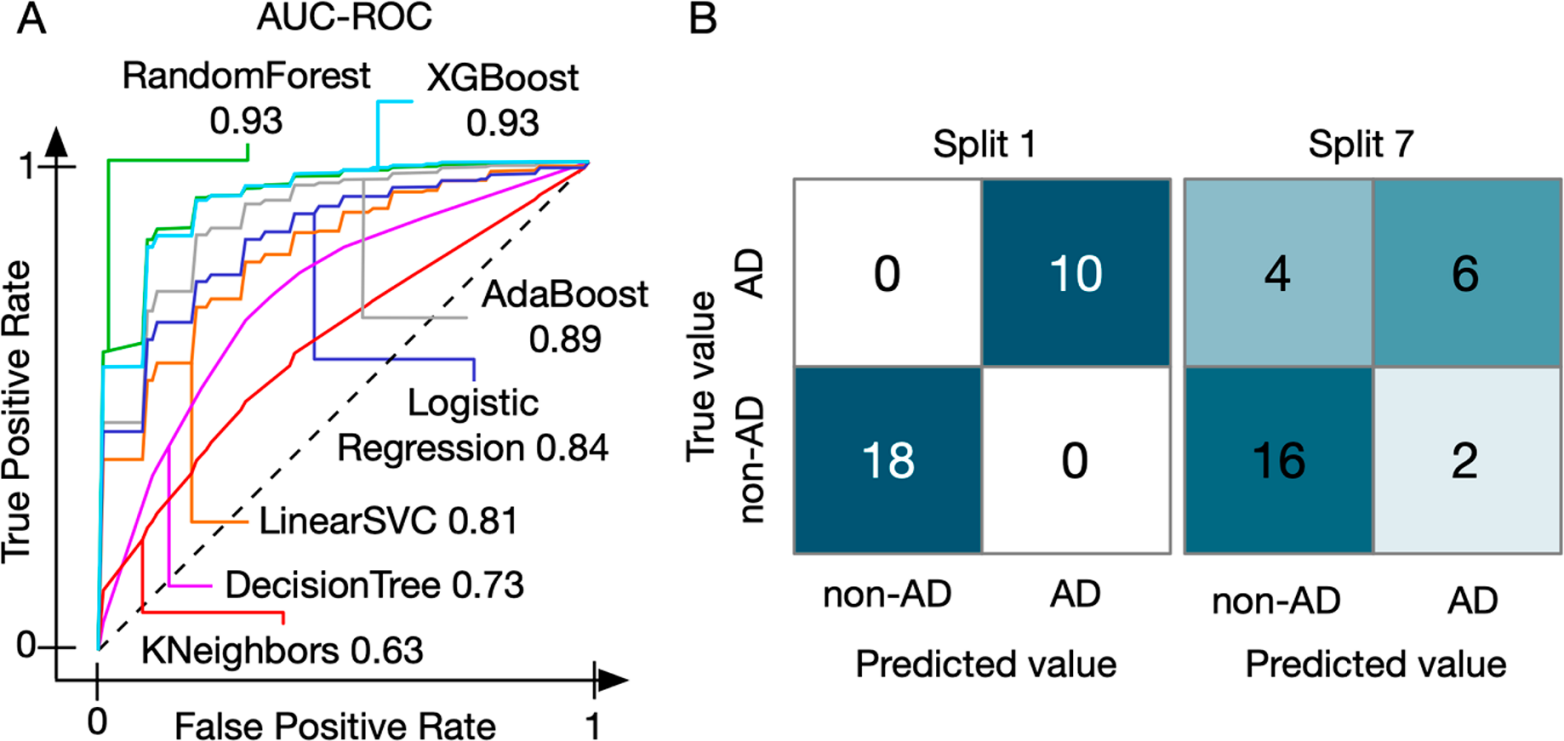
Application examples of OmicLearn. (A) ROC curves generated from the AD data set for multiple ML models. The achieved AUC-ROC ranged from 0.63 to 0.93. The different ML algorithms are indicated with their AUC-ROC value. (B) Examples of two different splits of the AD data set for one ML model. Split 1 resulted in perfect accuracy and exhibited no false classification. Split 7 of the same cross-validation run had several false classifications and hence lower performance, highlighting the importance of cross-validation.

Each model showed a different performance on predicting Alzheimer’s disease status. To illustrate such effects, we use several OmicLearn’s metrics in a “Run results for classifier” table and graphs to show the influence of classifiers (Figure 3A). The AUC-ROCs ranged from 0.63 to 0.93. This result cannot be due to differences other than the model as we had defined the same data subsets and other selections such as additional features and chose the same options for preprocessing, missing value imputation, feature selection, and cross-validation (Figure 2A). Further investigating the individual model performance highlights interesting characteristics. While the majority of the models achieve an AUC-ROC of larger than 0.8, there are some outliers with much lower performance such as KNeighbors with 0.63 ± 0.12 and the decision tree model with 0.73 ± 0.09. Interestingly, a rather simple model (LogisticRegression) that can serve as a baseline performance obtained an average AUC-ROC of 0.84 ± 0.09, which is higher than the more sophisticated support vector model (LinearSVC) with a mean score of 0.81 ± 0.09.

One of the best models for this application is the XGBoost classifier, which achieves an AUC-ROC of 0.93 ± 0.06. Note that the minimum AUC-ROC for a single CV split was 0.77, while the maximum was 1. This emphasizes that repeated validation is necessary to avoid misinterpreting performance on favorable or unfavorable splits.

A confusion matrix facilitates understanding performance metrics by showing actual numbers for each class (Figure 3B). To display the individual cross-validation splits, OmicLearn provides an interactive confusion matrix with a slider for picking a split. We even found perfect splits (e.g., split 1) that classified all Alzheimer’s patients (10/10) and non-Alzheimer’s patients (18/18) correctly. In contrast to that there are splits that are much worse (e.g., split 7), which only classify 6/10 and 16/18 correctly, highlighting the variance in prediction accuracy.

The described approach of having a baseline classifier, testing multiple classifiers, and characterizing results with multiple metrics aids in following community standards for machine learning in proteomics recommendations.^27^

## Discussion

Recent technological advances are dramatically improving robustness, throughput, and reproducibility of omics technologies such as genomics, proteomics, and metabolomics. This has sparked an increasing interest in using these technologies for biomarker discovery with large cohorts of clinical samples. More generally, the analysis and interpretation of large biological data sets obtained from omics technologies are complex and require automated computational workflows. In addition to the statistical tests that are typically applied, ML is an increasingly powerful tool to extract meaningful information and to obtain a deeper understanding of the underlying biology. The application of ML algorithms to large omics data sets, however, remains a challenge in many ways. Individual ML pipelines need to be established, specialized knowledge of data scientists or bioinformaticians is required, and the applied workflows often lack transparency and reproducibility. While the number of studies applying ML to omics data sets is rapidly increasing, issues associated with transparency of analyses, validation of existing results, and reproducibility are increasingly recognized and a matter of concern in the field.

To make ML algorithms easily accessible and their effects more understandable for experimental researchers, we developed OmicLearn, a browser-based app that allows applying modern ML algorithms to any omics data set uploaded in a tabular format. Although developed with clinical proteomics in mind, it is in no way limited to this application. OmicLearn offers several ways to explore the effect of a variety of parameter settings on ML performance and comes with a detailed documentation containing background information and a user manual. Within OmicLearn, multiple methods are available for preprocessing, feature selection algorithms, classification, and cross-validation steps together with hyperparameter tuning options so that existing results can be easily validated. Furthermore, OmicLearn enables researchers to export all settings and results as publication-ready figures with an accompanying methods summary. This enables researchers to apply the identical pipeline to multiple omics data sets or reproduce existing results and simplifies the application and usage of ML algorithms to any tabular data without requiring any prior ML knowledge. With its user-friendly interface, OmicLearn enables researchers to upload a data set with features, such as protein levels and any associated clinical information such as disease status, to train and test a model and provide new valuable insights into the data set. OmicLearn aggregates the methods and algorithms from the Python ML library scikit-learn together with XGBoost. Furthermore, it combines several best practices for CV to apply them to the files uploaded by users, such as MS-based proteomics data sets.

To demonstrate its usability, we have applied various ML algorithms to a recently published study that investigated changes in the CSF proteome of AD patients. While we showcased our app on proteomics data, it can be applied to tabular data obtained using other omics technologies such as genomics or metabolomics. A principal challenge that remains for all ML approaches is explainability. In a biomarker discovery context, features that give highly accurate models could originate from inherent study biases so that scrutinizing results with respect to the underlying biology is imperative. Therefore, before applying an ML layer with OmicLearn, users should have done previous EDA and have a good understanding of their data set. Even with sophisticated algorithms, a model can only be as good as the underlying data set.

A key finding is that ML requires repeated cross-validation of results as biased splitting of data can result in drastic performance variation, which can be larger than the performance difference of different classifiers. The interactive nature of OmicLearn aids in highlighting these differences. While some models will have better performance, the baseline classification accuracy of all classifiers should be in the same range and the user should be able to achieve competitive results with OmicLearn. This also suggests that it is beneficial to stringently benchmark a study with a relatively standard model (e.g., LogisticRegression) and have a good understanding of the baseline performance instead of purposely building a model for a particular study. In this way, OmicLearn also helps to democratize ML in the field as results will be more comparable and differences in model performance easier to understand.

In summary, OmicLearn is an easy-to-use, powerful tool to explore the application of ML algorithms. It gives a rapid overview of how well the supplied data perform in a classification task and can be applied to fine-tune and optimize or replicate ML models and highlight important features indicative of biomarkers. However, on its own, it does not provide biomarker panels or models ready to be used in diagnostics. The predictive power of the models should be critically questioned and ideally tested with independent cohorts. Furthermore, it does not include classical statistical methods such as analysis of covariance (ANCOVA). Potential improvements of OmicLearn include the diversification of ML classification algorithms and the inclusion of other sophisticated optimization and preprocessing methods such as standardization, imputation of missing values, and data encoding. We have found OmicLearn to be an effective tool to quickly analyze clinical proteomics data sets and hope that it will provide similar benefits for a large community of researchers in the field of biomarker discovery.

## Data Availability

The source code and data used can be found at https://github.com/MannLabs/OmicLearn, which also contains compiled one-click installers. Additionally, there are links to an online version of OmicLearn and a documentation hosted on ReadtheDocs.
